# Factors Influencing the Use of Tobacco Among Youth in Low-Income, Lower-Middle-Income, and Upper-Middle-Income Countries: A Systematic Review

**DOI:** 10.34172/jrhs.2024.152

**Published:** 2024-08-01

**Authors:** Fahad Ali Mangrio, Penpaktr Uthis, Suwimon Rojnawee

**Affiliations:** ^1^Nursing Student at the Faculty of Nursing, Chulalongkorn University, Bangkok, Thailand; ^2^Faculty of Nursing, Chulalongkorn University, Bangkok, Thailand

**Keywords:** Influential factors, Tobacco use, Youth, Low-income, Lower-middleincome, Upper-middle-income, Countries

## Abstract

**Background:** The use of tobacco is a significant global public health issue. According to the World Health Organization, tobacco use is a considerable risk factor for many diseases and causes more than 8 million deaths per year, with a disproportionate impact on low- and middle-income countries. Therefore, this systematic review was conducted to identify the factors influencing tobacco use among youth in low-income, lower-middle-income, and upper-middle-income countries.

**Study Design:** A system review.

**Methods:** The review followed the PRISMA guidelines, and the protocol was registered on PROSPERO (CRD42023430552). Several data sources were utilized, including PubMed, Scopus, ScienceDirect, MEDLINE, CINAHL, and ProQuest, and cross-sectional data from participants aged 15‒24 underwent investigation. Original full-text articles have been published between 2015 and 2023. Out of the 2892 studies, 20 were included in this review after two reviewers confirmed the eligibility criteria.

**Results:** The average age of the participants was (mean±standard deviation: 19.45±1.686). Most studies were conducted in lower-middle and upper-middle-income countries. Frequently reported influences were at the individual and social levels, including demographic, economic, and psychological parameters, attitude and knowledge, individual behavioral factors, parental education, family member tobacco use, stressful life events, and social networks. At the environmental level, factors included secondhand smoke exposure, community context, media channels, and access to tobacco.

**Conclusion:** The findings demonstrated a significant association between youth tobacco use and individual-, social-, and environmental-level factors. Consequently, specific interventions targeting these factors should be deployed to mitigate youth tobacco use in various socioeconomic settings.

## Background

 The use of tobacco among youth presents a substantial public health concern worldwide, with significant implications for their physical, mental, and social well-being.^1^ Tobacco use has primarily been defined as the consumption of tobacco products such as pipes, cigarillos, water pipes, locally grown tobacco, and smokeless tobacco products.^2^ Evidence has shown that approximately 80% of the global tobacco consumer population, which amounts to around 1.3 billion individuals, resides in low- and middle-income countries.^3^ The World Health Organization reported that tobacco use is responsible for six million deaths worldwide, with an additional 600 000 deaths attributed to secondhand smoke exposure.^4^ The frequency of tobacco use among youth differs across countries, and the overall prevalence of tobacco use is 19.33% across 133 low-income and middle-income countries.^5^ Tobacco use significantly decreases in high-income countries compared to low- and middle-income countries. For instance, in Australia, the prevalence of youth tobacco consumption has reduced from 7.0% to 3.0%^6^; in contrast, the prevalence of tobacco use among youth in low- and middle-income countries, such as Madagascar (23.75%),^5^ Nepal (20.5%), and Haiti (19.75%), has demonstrated an increase.^7^ In addition, a systematic review found extensive variations in tobacco use proportion, ranging from 2% to over 30%, depending on the geographical location and specific tobacco products.^8^

 Tobacco use is a significant risk factor for various chronic diseases, such as cancer, cardiovascular diseases, mental health issues, and respiratory diseases.^6^ The burden of morbidity and mortality from these diseases is strongly linked with tobacco use in low-income, lower-middle-income, and upper-middle-income countries.^8^ Moreover, tobacco consumption impacts the spread of poverty and inequality by diverting household resources away from essential needs, subsequently reducing productivity and income, and increasing healthcare expenditures.^9^ Further, tobacco use has harmful environmental impacts that adversely affect the livelihood and well-being of individuals in low- and middle-income countries.^10^

 The World Bank ranks world economies into four group classes of income in July each year based on gross national income per capita.^11,12^ The present group classification identifies 26 countries as low-income and 108 as middle-income, further divided into 60 upper-middle-income and 48 lower-middle-income countries.^11^ Overall, these countries account for approximately 75% of the global population and contribute to 40% of the world’s economic activity.^12^ These outcomes emphasize the necessity of coordinated attempts to reduce tobacco consumption among youth in low-income, lower-middle-income, and middle-income countries.

 The youth populations are specifically prone to engaging in tobacco use due to the influence of numerous factors such as peer pressure, social norms, and targeted marketing schemes used by the tobacco industry.^13^ The early initiation of tobacco use has profound effects on both physical and cognitive development.^14^ Individuals who initiate tobacco smoking at an early age are more prone to developing tobacco addiction and face greater challenges when struggling to quit smoking in adulthood.^15^ A further systematic review suggested that most studies were conducted in high-income countries, despite youth smoking rates being substantially higher in lower- and middle-income countries.^16^ Moreover, understanding the socioeconomic determinants of youth tobacco smoking is crucial for effective interventions.^17^ Therefore, it is necessary to conduct research on factors affecting tobacco use within diverse socioeconomic contexts to develop tailored interventions and address the consequences of tobacco-related diseases and disparities. Significantly, based on the current body of knowledge, this systematic review represents the first comprehensive examination of factors associated with tobacco use among youth in low-income, lower-middle-income, and upper-middle-income countries.

 The primary objective of this systematic review was to investigate factors influencing tobacco use among youth in low-income, lower-middle-income, and upper-middle-income countries.

## Methods

###  Study design

 This systematic review followed the guidelines outlined in the Preferred Reporting Items for Systematic Reviews and Meta-Analysis (PRISMA).^18^ The review adhered to the 27-item checklist and revised the flowchart provided in the PRISMA guidelines. The protocol for this systematic review was registered in PROSPERO (CRD42023430552).

###  Population, exposure, comparison, outcome, and study design criteria

*Population:* It included individuals who fall within the age range of 15‒24, as defined by the United Nations youth definition,^19,20^ and who have used any form of tobacco (i.e., cigarettes, cigars, smokeless tobacco, hookah, heated tobacco products, nicotine pouches, or e-cigarettes) within the last 30 days. Additionally, these individuals reside in countries that fall under the classification of low-income, lower-middle-income, or upper-middle-income, as determined by the World Bank.^12^

*Exposure:* Influencing factors.

*Comparison:* No.

*Outcomes:* Tobacco use among youth.

*Study design:* Cross-sectional.

###  Selection criteria

 The study was based on original research and available full texts. To ensure the inclusion of the most up-to-date findings and credible evidence for timely decisions, the publication period of studies for this systematic review was restricted from 2015 to 2023.^21-23^

###  Search strategy

 Multiple databases, such as PubMed, Scopus, ScienceDirect, MEDLINE, CINAHL, and ProQuest, were extensively searched using generated MeSH (Medical Subject Headings) terms and keywords.They included factors influencing OR predictors influencing OR identifying factors OR associated factors AND tobacco abuse OR cigarette consumption OR chewing tobacco OR smokeless tobacco, AND youth OR young people OR adolescents. After searching, the studies were selected in accordance with the 2022 World Bank Index of Economic Classifications,^12^ which encompasses countries with low-income, lower-middle-income, and upper-middle-income status. Moreover, the investigators formulated and implemented search methodologies under the guidance of a skilled health science librarian.

###  Study selection

**Figure 1:**
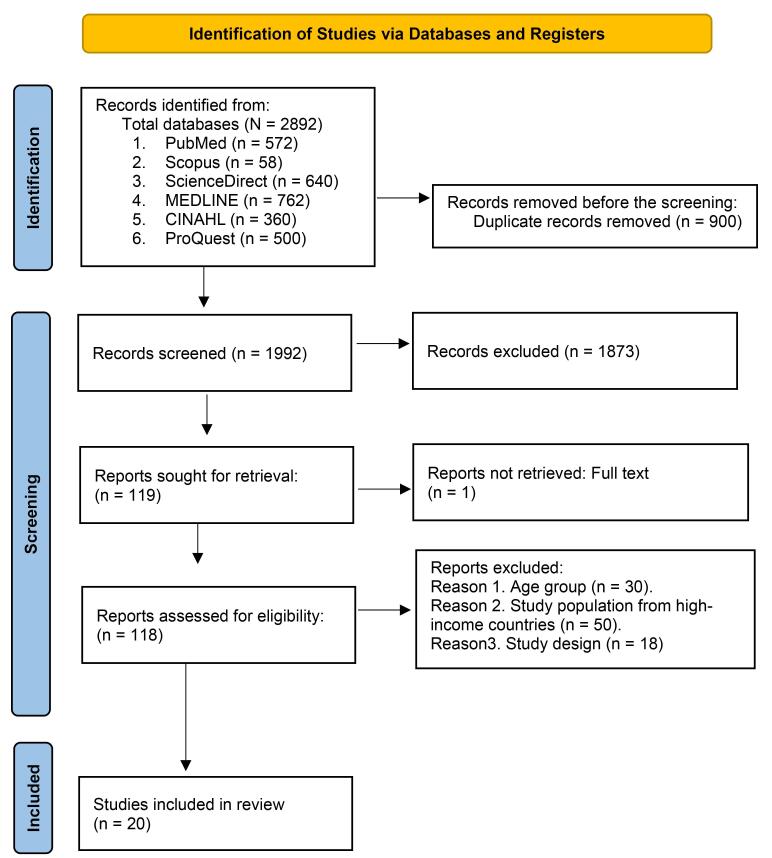
PRISMA flow chart. *Note*. PRIMA: Preferred Reporting Items for Systematic Reviews and Meta-Analyses

 The initial search across databases yielded 2892 articles (Figure 1). Two independent reviewers screened the titles and abstracts of potential articles to determine the eligibility criteria. If an article potentially met the inclusion criteria, the complete article was obtained and reviewed to confirm eligibility and inclusion in the review. Disagreements between reviewers were resolved through discussion or the involvement of a third reviewer when necessary.

 Nine hundred duplicate articles were removed using the EndNote 21 “find duplicate” library feature and manual inspection. During the title and abstract screening process, 1873 records were excluded based on failure to meet study eligibility criteria. Of these, 500 articles did not address factors associated with youth tobacco use, and the remaining 1373 articles were not specific to the study objective. In addition, one article did not retrieve the full text.^24^ Next, full-text versions were obtained for the remaining 118 articles. Two authors (F. A. M. and S. R.) independently reviewed these articles based on the inclusion and exclusion criteria. After the full-text review, 20 articles were determined to be eligible and included in this systematic review.

###  Data extraction

 The data extracted from these studies were synthesized and summarized using the matrix method^25^ and the narrative synthesis technique.^26^ Table 1 presents a comprehensive summary of the included 20 studies, including author(s) name and year of publication, country economic status, age range, study design, sample size, measurement, prevalence of smoking and smokeless tobacco use, identified risk factors, statistical findings, and quality appraisal scores. Furthermore, all the text from the result summary was coded line-by-line for reported influences on tobacco use under individual-, social-, and environmental-level factors based on an adapted model of the Theory of Triadic Influence.^27^

**Table 1:** Summary of the data from all eligible included studies

**Author(s) name and year of publication**	**Country economic status**	**Age range**	**Study** **design**	**Sample** **size**	**Measurement**	**Prevalence of smoking and smokeless tobacco use**	**Identified risk factors**	**Statistical findings** **OR (95% Cl)**	**Quality appraisal scores**
**Upper-middle-income countries **									
Othman et al(2017) ^28^	Iraq	18-24	Cross-sectional	1,160	Self-report	Cigarette (10.0%) and waterpipe (28.0%)	Male gender	5.70 (3.90, 8.20)	Strong
							Drink alcohol and other substances	2.80 (1.40, 5.60)	
Chirtkiatsakul et al(2019) ^29^	Malaysia	18-24	Cross-sectional	843	Self-report	Cigarette (22.4%)	Male gender	12.60 (8.04, 19.89)	Moderate
							Mothers with primary-level education	4.02 (1.40, 11.50)	
Favorable attitudes toward smoking	9.09 (4.98, 16.50)
Equivocal attitudes	2.98 (1.78, 5.00)
Suwanwong et al (2021)^30^	Thailand	15-19	Cross-sectional	6,046	Self-report	Cigarette (6.4%)	Male gender	34.29 (16.60, 70.73)	Strong
							Alcohol use	17.05 (12.87, 22.59)	
							Exposure secondhand smoke home	6.63 (4.98, 8.82)	
							Exposure secondhand smoke school	2.02 (1.38, 2.97)	
							Exposure secondhand smoke at restaurant	1.57 (1.17, 2.10)	
							Public transport	1.42 (1.02, 1.98)	
							Alcohol use	17.05 (12.87, 22.59)	
							Tobacco advertising	1.90 (1.31, 2.75)	
Yusof et al (2019)^31^	Malaysia	18-19	Cross-sectional	388	Self-report	E-cigarette (14.4%)	Gender male	25.70 (10.88, 60.71)	Strong
							Peer use of e-cigarettes	19.93 (11.18, 35.55)	
Todorović et al (2022)^32^	Bosnia & Herzegovina	18-24	Cross-sectional	1,200	Self-report	Cigarette (34.1%)	Secondhand smoke at home	1.19 (1.09, 1.30)	Strong
							More money available	1.19 (1.05, 1.34)	
Gazibara et al (2021)^33^	Kosovo, Serbia	17-21	Cross- section	514	Self-report	Cigarette (22.6%)	Exposure secondhand smoke	1.07 (1.01, 1.13)	Strong
							Severe depressive symptoms	1.12 (1.07, 1.18)	
							Living with smokers	3.78 (1.69, 8.07)	
							Alcohol consumption	2.98 (1.19, 10.03)	
Ninkron et al (2022)^34^	Thailand	15-18	Cross-sectional	290	Self-report	Cigarette (51.5%)	Parents deceased	2.28 (1.21, 3.40)	Strong
							Divorced parents	1.60 (1.15, 2.50)	
							Poor academic level	2.50 (1.13, 3.55)	
							More money available	1.19 (1.05, 1.34)	
							Severe depressive symptoms	1.12 (1.07, 1.18)	
							Living with smokers	3.78 (1.69, 8.07)	
							Alcohol consumption	2.98 (1.19, 10.03)	
**Lower-middle-income countries**									
Odukoya et al (2016)^35^	Nigeria	15-24	Cross-sectional	326	Self-report	Cigarette (32.5%) Chewing tobacco (21.4%)	Living with friends	1.98 (1.01, 3.86)	Strong
							Alcohol drinking	6.16 (3.03, 12.54)	
							Gender (male)	2.56 (1.83, 7.80)	
Lalithambigai et al (2016)^36^	India	18-20	Cross-sectional	720	Self-report	Cigarette (20.4%)	Male gender	8.50 (3.26, 22.50)	Moderate
							Peer’s smoking	5.15 (2.21, 11.90)	
							Daily pocket money	4.16 (1.76, 9.82)	
Bigwanto et al (2017)^37^	Indonesia	15-19	Cross-sectional	690	Self-report	Cigarette (29.6%)	Gender (male)	31.80 (17.57, 57.63)	Strong
							Cigarette advertising	1.24 (0.89, 1.73)	
							Availability	3.72 (2.62, 5.28)	
Kristina et al (2020)^­­­38^	Indonesia	16-24	Cross-sectional	920	Self-report	E-cigarette (10.68%) Cigarette (57.61%)	Gender (male)	2.32 (1.23, 3.45)	Strong
							Alcohol consumption	2.35 (1.56, 3.89)	
							Poor smoking knowledge	1.92 (1.32, 3.22)	
							Smoking attitudes (Neutral)	1.87 (1.31, 3.15)	
Fauzi andAreesantichai ‎ (2020)^39^	Indonesia	15-19	Cross-sectional	1,318	Self-report	E-cigarette (36.3%)	Gender (male)	3.52 (2.31, 5.35)	Strong
							Peer’s use of e-cigarette	2.07 (1.31, 3.27)	
							Easy availability	2.37 (1.52, 3.67)	
							School location	1.71 (1.15, 2.50)	
Tucktuck et al (2017)^40^	Palestine (west bank and Gaza strip	18-24	Cross-sectional	1,891	Self-report	Waterpipe (24.4%) Cigarette (18.0%)	Male gender	10.91 (7.20, 16.40)	Moderate
							Rural areas	1.90 (1.17, 3.00)	
							Living without family	1.70 (1.19, 2.68)	
							High financial status	1.60 (1.24, 2.20)	
							Low academic achievement	4.50 (2.78, 7.30)	
Baheiraei et al (2016)^41^	Iran	15-18	Cross-sectional	870	Self-report	Cigarette (20.3%)	Availability of tobacco	2.90 (1.90, 4.20)	Strong
							Friends’ use of drugs	3.40 (2.00, 6.00)	
							Intention to use tobacco	2.60 (1.40, 4.70)	
							Interaction with antisocial peers	3.40 (1.30, 8.70)	
							Poor family support	2.30 (1.60, 3.30)	
							Family conflict	1.80 (1.20, 2.60)	
							Academic failure	3.00 (2.10, 4.20)	
Efendi et al (2021)^42^	Indonesia	15-24	Cross-sectional	4,811	Self-report	Cigarette (54.6%)	Low educational level	1.93 (1.52, 2.45)	Strong
							Exposure to tobacco by radio	1.28 (1.12, 1.48)	
							Age 20-24 years	2.80 (2.40, 3.20)	
Grover et al (2020)^43^	India	15-24	Cross-sectional	13,329	Self-report	Cigarette (5.0%) Smokeless (10.9%)	Residence (rural)	1.30 (1.20, 1.54)	Moderate
							Unmarried	1.50 (1.37, 1.70)	
Akhter et al (2018)^44^	Pakistan	18-24	Cross-sectional	689	Self-report	Cigarette (13.72%) Waterpipe (49.01%)	Social gatherings	5.03 (1.18, 21.40)	Moderate
							Reduce stress wants to relax	1.72 (1.03, 2.86)	
							No anti-smoking awareness on social media	2.22 (1.24, 3.99)	
Rahman and Tareque, 2020^45^	Bangladesh	15-24	Cross-sectional	385	Self-report	Cigarette (40.3%)	Low monthly income	7.97 (1.68, 37.70)	Strong
							Father’s smoking	2.5 (1.39, 4.50)	
							Brother’s smoking	2.88 (1.39, 5.96)	
							Friend’s smoking	9.85 (5.85, 15.27)	
**Low-income countries**									
Duko et al (2019)^46^	Ethiopia	15-22	Cross-sectional	564	Self-report	Cigarette (11.0 %)	Friends’ smoke	4.00 (2.04, 7.40)	Strong
							Alcohol drinking	4.10 (1.84, 9.70)	
							Illicit drug use	5.80 (1.90, 17.30)	
Kubas and Wadi (2015)^47^	Yemen	18-24	Cross-sectional	480	Self-report	Cigarette (2.4%) water pipe (78.6%)	Curiosity	4.8%	Week
							Friends’ smoking	9.5%	
							Enjoyment	14.3%	
							Life stress	9.5%	

*Note*. OR: Odds ratio; CI: Confidence interval.

###  Quality appraisal assessment

 The authors systematically assessed the methodological quality of all 20 eligible studies using the Joanna Briggs Institute Meta-Analysis Assessment and Review Instrument.^48^ Critical appraisal tools for cross-sectional studies were used to evaluate the methodological quality of eligible studies. This tool covers eight components, including the study objectives, inclusion criteria, information about study participants and setting, measurement quality, identification and management of potential confounding variables, and statistical analyses. First, the eight components were rated on a three-point scale (1 = strong, 2 = moderate, or 3 = weak) based on the defined quality rating criteria.^49^ An overall “strong” rating was defined as having no weak and at least six strong ratings. An overall “moderate” rating was described as one weak and less than six strong ratings. A “weak” rating was warranted if two or more weak ratings were across the eight evaluation components.^48^

## Results

 Out of the 2,892 articles identified, 20 met the study eligibility criteria. Studies included in the review used a cross-sectional design. The average age (M ± standard deviation) was 19.45 ± 1.686. The sample size ranged from 290 to 13,329 (M = 2,054). The majority of studies were conducted in lower-middle-income countries (n = 11), followed by upper-middle-income countries (n = 7) and low-income countries (n = 2)

**Figure 2:**
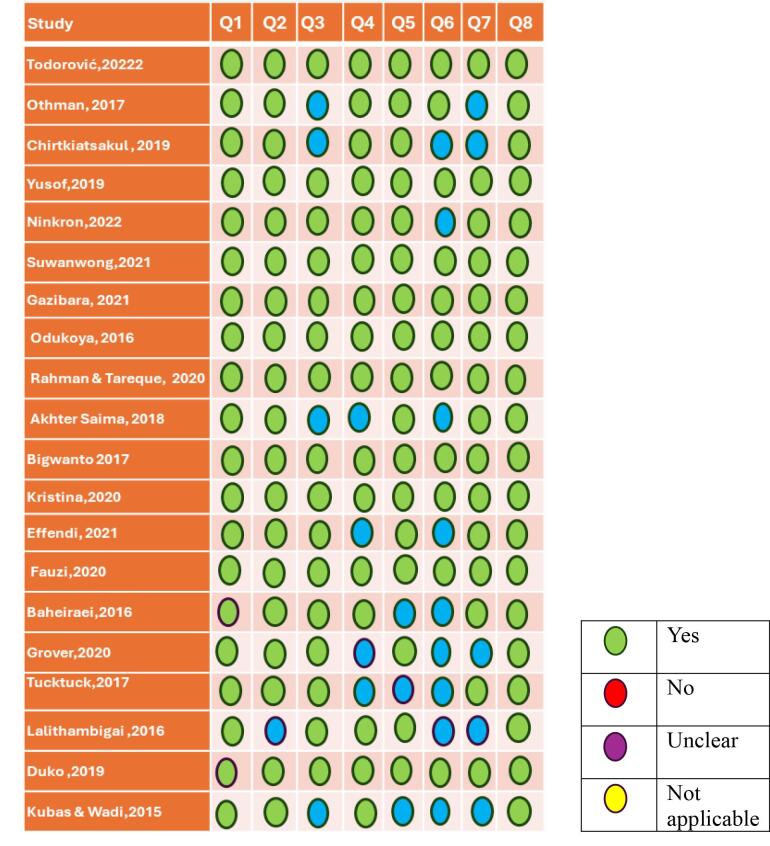
JBI Checklist for the Quality Assessment of Cross-sectional Studies. Q1. Were the criteria for inclusion in the sample clearly defined? Q2. Were the study subjects and the setting described in detail? Q3. Was the exposure measured in a valid and reliable way? Q4. Were objective, standard criteria used for the measurement of the condition? Q5. Were confounding factors identified? Q6. Were strategies to deal with confounding factors stated? Q7. Were the outcomes measured in a valid and reliable way? Q8. Was the applied statistical analysis appropriate?

 According to the quality appraisal evaluation (Figure 2), out of the eligible studies, fourteen^28,30-35,37-39,41,42,45,47^ were rated as strong, while five^29,36,40,43,44^ were rated as moderate in methodological rigor. Therefore, many studies had sound research designs and methodologies, which can lead to dependable and valid outcomes. However, one study^46^ received a weak rating, revealing areas where their methodologies could be enhanced to boost their overall quality and dependability.

###  Measurement and prevalence of youth tobacco use

 All studies employed self-reporting techniques to evaluate current tobacco consumption. In upper-middle-income countries, tobacco smoking rates ranged from 6.4% to 51.5%, with different modes of consumption, such as cigarette smoking, e-cigarettes, and water pipe use.^28-34^ Meanwhile, tobacco use in lower-middle-income countries^35-45^ fluctuated between 5.0% and 57.6%, with various forms of consumption, such as cigarette smoking, e-cigarettes, water pipe use, smokeless tobacco, and chewing tobacco. Among youth in low-income countries,^46,47^ cigarette smoking ranged from 2.4% to 11.0%, with water pipe use being the most prevalent at 78.6%.

###  The main findings reported on the theory of triadic influence

 Across all included studies, the most reported influences were on individual- (n = 18) and social- (n = 13) level factors, and fewer studies measured environmental-level factors (n = 10). The main findings are outlined in Table 2 and discussed later, according to the country’s level.

**Table 2:** Summary of factors influencing tobacco use among youth based on the theory of triadic influence

**Individual-level factors **
*a. Demographic *
Gender (Male)
Age
Unmarried
Living without family
*b. Economic factors*
Low monthly income Financial status (High) Daily pocket money
*c. Psychological factors*
Coping stress Curiosity Depression symptoms Intention to tobacco use Enjoyment
*d. Attitude and knowledge *
Low education level Favorable attitude toward smoking Equivocal attitude
*e. Individual behavior *
Alcohol consumption Illicit drug use
**Social level factors **
*a. Parents education *
*Mothers with primary education*
*b. Family member’s tobacco use *
Father smoking Mother smoking Brother smoking
*c. Stressful life events *
Death of parents Divorce of parents Poor academic performance Life stress Poor family support Family conflict
*d. Social networks *
Social gathering Peers smoking Living with friends Interaction with antisocial peers
**Environmental-level factors **
*a. secondhand smoke exposure*
Secondhand smoke at home, public transport, and living in a smoker’s environment
*b. Community context *
Rural area School location
*c. Media channels *
Smoking advertising Exposure to tobacco by radio
*d. Access of tobacco *
Easy availability

###  Factors influencing tobacco use in upper-middle-income countries

*Individual-level factors:* This included demographics, economic status, psychological parameters, attitudes, and knowledge, as well as individual behavioral factors; four studies^28-31^ revealed that males are more prone to tobacco use than females. One study^32^ demonstrated that the high availability of money was also a significant factor in tobacco use. The findings of another study^33^ showed severe depressive symptoms, and one study^29^ showed that favorable or equivocal attitudes were positively associated with tobacco use. Three studies^28,30,33^ found that alcohol consumption and other illicit drugs increased the likelihood of tobacco use among youth.

*Social-level factors:* They consisted of parents’ education, peers’ tobacco use, and stressful life events. One study^29^ revealed that mothers with primary-level education increased the likelihood of tobacco use among youth. Another study^31^ indicated that the use of electronic cigarettes by peers increased the likelihood of tobacco use among youth.Further, one^34^ showed poor academic performance, and another^34^ indicated that family circumstances, such as divorced parents and the death of one or both parents, increased the likelihood of tobacco use.

*Environmental factors:* Three studies^30,32,33^ reported that secondhand smoke exposure is linked to higher rates of tobacco use among youth across various settings such as homes, restaurants, schools, public transport, or living with smokers.

###  Factors influencing tobacco use in lower-middle-income countries

*Individual factors.* Various demographic, economic, psychological, attitude and knowledge, and individual behavioral factors were found to be positively correlated with tobacco use among youth. These factors were included in six studies^35-40^ on gender (male), three studies^40-42^ on low education level, and three studies^40,43,44^ that have shown that unmarried individuals and those living away from their families may have fewer social constraints and are more likely to engage in tobacco. One study^45^ revealed low monthly income, and two studies^36,40^ showed that high financial status significantly increased the likelihood of tobacco use. One study^38^ demonstrated poor knowledge and neutral attitudes about smoking harm. Further, another study^44^ reported relaxation and coping with stress. Moreover, two studies^38,44^ showed that they intended to use tobacco and alcohol.

*Social-level factors:* They included family members smoking, stressful life events, and social networking.One study^45^ indicated that father and brother’s tobacco smoking was associated with tobacco use among youths. Further, another study^41^ indicated that family conflict and poor family support also increase the likelihood of tobacco smoking. Four studies^36,39,41,45^ represented that peer tobacco smoking and other illicit drug use were associated with tobacco use among youth. Additionally, three studies demonstrated that interaction with antisocial peers,^41^ living with friends,^35^ and social gatherings^44^ among youth had a positive association with tobacco use.

*Environmental-level factors:* They encompassed media channels, community context, and access to tobacco. Two studies revealed that exposure to tobacco smoking by radio^42^ and smoking advertising^37^ increases tobacco use. In contrast, one study^44^ reported that a lack of anti-smoking information on social media and traditional media is associated with its use among youth. Moreover, other studies showed that exposure to smoking in school locations^41^ and living in rural areas^40,43^ increased the likelihood of tobacco consumption among youth. Furthermore, three studies^37,39,41^ found that the easy availability of tobacco in markets and communities increased the likelihood of tobacco use among youth.

###  Factors influencing tobacco use in low-income countries

*Individual-level factors:* They consisted of psychological and behavioral parameters. One study^47^ indicated that curiosity and enjoyment were significantly associated with tobacco smoking among youth. Additionally, a study^46^ confirmed that the use of illegal drugs and alcohol consumption increased the likelihood of tobacco smoking among youths.

*Social-level factors:* They included social networks and stressful life events.Two studies^46,47^ reported that tobacco-smoking friends played an important role in tobacco use among youth. One study^47^ revealed that life stress was significantly associated with tobacco smoking among youth.

## Discussion

 This systematic review explored factors influencing tobacco use among youth in low-, lower-, and upper-middle-income countries. The review included 20 studies after narrowing down potentially relevant studies for the full text and considering the inclusion criteria. All included studies used cross-sectional designs that met the eligibility criteria for the review. The overall study quality varied from weak to strong. The primary factors that compromised methodological rigor were approaches utilized to address the confounding factors. Addressing potential confounding factors can be achieved using restriction, matching, and statistical adjustment methods.^50^ For instance, when investigating the correlation between factors influencing youth tobacco use, validity can be improved by limiting the study population, matching participants with similar characteristics, and employing statistical techniques such as regression to manage confounding variables.^48^ There was variation in the prevalence of tobacco use among different income categories of countries, with higher rates observed in lower-middle-income countries.

 Studies included in the review investigated factors associated with three identified domains, namely, individual-, social-, and environmental-level factors. One individual-level factor was demographics. Male gender was identified as a significant influencing factor in lower- and upper-middle-income countries.^28-31,35-40^ According to the global gender gap report 2022,^51^ men globally show a lower healthy life expectancy than women, apart from Sub-Saharan Africa. Furthermore, men are at a higher risk of mortality due to non-communicable diseases, such as cardiovascular diseases, cancers, respiratory diseases, and diabetes, than women. Therefore, it is suggested that health policies and programs that consider the unique needs and preferences of both men and women be promoted and implemented in the future.

###  Socioeconomic status

 There was an inconsistent relationship between financial status and tobacco use among youth in lower-middle-income countries. In two studies,^36,40^ higher income or daily pocket money was associated with increased tobacco use, whereas one study^45^ found that lower income was also significant. This may reflect the various levels of affordability and accessibility of tobacco products in different settings and the different motivations and influences of tobacco use among youth.

###  Psychological and behavioral factors

 Studies^29,41,47^ conducted in low-income, lower-income, and upper-middle-income countries proved a positive association between favorable attitude, intention, and curiosity toward tobacco use. Earlier systematic reviews and meta-analyses revealed a positive association between favorable attitudes, intentions, and curiosity toward tobacco use among adolescents.^52^ Furthermore, studies^44,47^ from low- and lower-middle-income countries demonstrated that stress coping levels and relaxation had a significant relationship with tobacco use among youth. These findings underscore the importance of implementing comprehensive programs focusing on tobacco prevention and providing youth with effective stress-coping mechanisms and relaxation skills. In addition, regarding substance use, one study from low-income countries and another from upper-middle-income countries confirmed a positive association between tobacco use and individuals who were already consuming alcohol and using other illicit drugs.^28,47^ In an earlier review of a multi-country analysis, tobacco use was found to be influenced by alcohol consumption and other illicit drug use.^53^ This study indicated an explicit relationship between tobacco consumption, alcohol consumption, and other illicit drug use in low- and middle-income countries.

###  Social factors

 Family impacts are essential components of the tobacco use behavior of youth.^54^ This study explicitly identified a significant relationship between tobacco use and factors influencing father and brother’s tobacco use and family conflicts in lower-middle-income countries.^45^ In comparison, a previous systematic review consistent with 41 studies in 20 low- and middle-income countries represented compelling evidence of an association between parental and sibling tobacco smoking and tobacco use among youths. Furthermore, the study also indicated that family conflicts, low parental monitoring, and poor parent-child communication are factors that affect youth tobacco use.^55^ Moreover, one study in an upper-middle-income country found that the lower education of mothers significantly increased the likelihood of tobacco use among youth.^29^ A previous review of education and tobacco use suggested that education may reduce tobacco consumption.^55^ Peer influence on tobacco use and other types of illicit drug use among youth was significant in two low-income countries^46,47^ and four lower-middle-income countries.^36,39,41,45^ Similarly, one study showed that peer tobacco use has a positive relationship with youth tobacco use.^53^

###  Environmental factors

 Two studies^42,44^ conducted in lower-middle-income countries revealed that not using social media and traditional media, such as newspapers or television, and a lack of awareness about the harms of tobacco were positively associated with tobacco use. In contrast, an earlier survey performed among adolescents in 10 low-income countries in Africa and Asia has proven a positive correlation between tobacco use and exposure to tobacco advertising and promotion across multiple mass media platforms. Another study showed a negative correlation between tobacco use and awareness of the detrimental effects of tobacco use, as well as exposure to mass media anti-tobacco messages.^56^

 It is essential to acknowledge the limitations of this systematic review when interpreting the findings. The major limitation of this study is the need for more generalizability on the youth of high-income countries because the studies consisted of only low-income, lower-income, and upper-middle-income countries. In addition, publication bias may occur due to the study period being limited between 2015 and 2023, which excludes studies published before this time limit. Furthermore, studies were excluded based on the definition of youth age. However, it is essential to consider the limitations of the studies with moderate and weak ratings, as they may have flaws in their methods or analyses.

 This systematic review suggests that tobacco education programs should focus on mothers’ education levels. Further, it is important to address individual-, social-, and environmental-level factors such as male gender and psychological issues, as well as the influence of social networks, including peers, across various settings. Tailored interventions such as peer support, social norm change, and awareness campaigns that adhere to strict marketing regulations through social and traditional media should be explored for their effectiveness.

**Highlights**

- This review analyzed 20 cross-sectional studies focusing on factors associated with youth tobacco use in low-income, lower-middle-income, and upper-middle-income countries.
- Three overarching domains emerged based on the theory of the triadic influence model, namely, Individual-, social-, and environmental-level factors.
- Individual-level factors included demographic, economic, psychological, attitude, knowledge, and individual behavioral factors.
- Social-level factors were parental education, family member tobacco use, stressful life events, and social networks.
- Environmental-level factors encompassed secondhand smoke exposure, community context, media channels, and access to tobacco.

## Conclusion

 The findings are categorized into individual, social, and environmental domains. Individual-level factors include demographic, economic, psychological, attitude and knowledge, and individual behavioral factors. Social-level factors encompass parental education, family member tobacco use, stressful life events, and social networks. Environmental factors include secondhand smoke exposure, community context, media channels, and access to tobacco. These factors were found to be significantly associated with tobacco use among youth in low-, lower-, and upper-middle-income countries. Consequently, it is imperative to implement targeted, tailored health interventions that aim to reduce tobacco use among individuals in diverse socioeconomic contexts.

## Acknowledgments

 This systematic review is part of a Ph.D. nursing dissertation submitted by Fahad Ali, supported by the Graduate ASEAN Non-ASEAN Countries Scholarship program at the Faculty of Nursing, Chulalongkorn University in Bangkok, Thailand.

## Authors’ Contribution

**Conceptualization:** Fahad Ali Mangrio.

**Data curation:** Fahad Ali Mangrio, Suwimon Rojnawee.

**Formal analysis:** Fahad Ali Mangrio, Suwimon Rojnawee.

**Funding acquisition:** Penpaktr Uthis.

**Investigation:** Fahad Ali Mangrio, Suwimon Rojnawee.

**Methodology:** Penpaktr Uthis, Fahad Ali Mangrio.

**Project administration:** Penpaktr Uthis.

**Resources:** Penpaktr Uthis.

**Software:** Fahad Ali Mangrio.

**Supervision:** Penpaktr Uthis.

**Validation:** Fahad Ali Mangrio, Penpaktr Uthis.

**Visualization:** Fahad Ali Mangrio, Suwimon Rojnawee.

**Writing–original draft:** Fahad Ali Mangrio.

**Writing–review & editing:** Fahad Ali Mangrio, Suwimon Rojnawee.

## Competing Interests

 The authors have no conflict of interests to declare for this study.

## Ethical Approval

 This study included no human participants, and no ethical review was performed for it

## Funding

 This research received no external funding.
